# Impact of idiopathic short stature on children’s well-being: a nationwide study of short Danish children

**DOI:** 10.1210/jendso/bvag038

**Published:** 2026-02-26

**Authors:** Anders Juul, Agnès Linglart, Michael Højby, Tina Lund Leunbach, Alberto Pietropoli, Kasper Løwe Lundgren, Lasse de Fries Jensen

**Affiliations:** Department of Growth and Reproduction, Copenhagen University Hospital–Rigshospitalet, Copenhagen 2100, Denmark; International Centre for Research and Research Training in Endocrine Disruption of Male Reproduction and Child Health (EDMaRC), Copenhagen University Hospital–Rigshospitalet, Copenhagen 2100, Denmark; Department of Clinical Medicine, Copenhagen University Hospital–Rigshospitalet, Copenhagen 2100, Denmark; Endocrinology and Diabetes Department, Bicêtre Paris Saclay Hospital, Le Kremlin-Bicêtre 94270, France; Novo Nordisk A/S, Clinical Development, Søborg 2860, Denmark; Novo Nordisk A/S, Clinical Development, Søborg 2860, Denmark; Novo Nordisk Healthcare AG, Zürich 8058, Switzerland; EY-Parthenon, Frederiksberg 2000, Denmark; Novo Nordisk A/S, Clinical Development, Søborg 2860, Denmark

**Keywords:** idiopathic short stature, psychosocial functioning, growth hormone, registries, pediatrics

## Abstract

**Context:**

It remains unclear whether idiopathic short stature (ISS), defined as a height below −2.0 SD score (SDS) without an identifiable cause, adversely affects psychosocial well-being.

**Objective:**

To assess the psychosocial impact of ISS in children using nationwide registry and survey data.

**Design:**

The nationwide cohort study included children identified between 2012 and 2020 with follow-up questionnaire data up until 2022.

**Setting:**

School-aged children in Denmark.

**Patients or Other Participants:**

The National Child Health Register contained heights on 507 754 children of which 42 185 had heights below −2.0 SDS. Children with a diagnostic code associated with potential underlying causes for short stature in the Danish National Patient Registry were excluded. The final cohort included 16 121 children with ISS. Each child was matched to 5 controls by age, sex, and region.

**Intervention(s):**

None.

**Main Outcome Measure(s):**

Psychosocial well-being (bullying, loneliness, and insecurity) assessed by mandatory National Well-being Surveys (2015-2022). Odds ratios (ORs) were calculated across school grade levels.

**Results:**

Children with ISS had higher odds of feeling bullied (school grade levels 0-3: OR 1.03 [95% confidence interval (CI) 0.95-1.12]; 4-6: OR 1.37 [95% CI 1.24-1.56]; 7-9: OR 1.19 [95% CI 1.02-1.40]), lonely (0-3: OR 1.02 [95% CI 0.93-1.12]; 4-6: OR 1.15 [95% CI 1.06-1.27]; 7-9: OR 1.11 [95% CI 1.01-1.25]), and insecure (0-3: OR 1.08 [95% CI 1.00-1.17]; 4-6: OR 1.07 [95% CI 1.02-1.15]; 7-9: OR 1.09 [95% CI 1.04-1.19]).

**Conclusion:**

Children with ISS displayed increased psychosocial distress. These findings support psychosocial assessment in children with ISS to enable timely intervention.

Short stature in a child is a common cause of referral for further evaluation by pediatric endocrinologists [1]. Short stature is defined as a height of at least 2 SD scores (SDS) below the corresponding mean height for a given age, sex, and population [2]. The incidence of short stature is estimated at 23 per 1000 children [3]. In most children presenting with short stature, an underlying cause cannot be identified [2]. When systemic, endocrine, nutritional, or chromosomal abnormalities or known genetic variants have been ruled out, cases have traditionally been classified as idiopathic short stature (ISS) [2]. Genetic tests should be undertaken depending on clinical findings; eg, in case of mesomelia, SHOX analysis may be relevant [2]. The advancements in systematic phenotyping, targeted gene panels, and whole-exome sequencing have enabled the identification of underlying diagnoses in 25% to 40% of cases previously classified as ISS [4].

Short stature is considered a problem as it may be perceived as a disability and may furthermore be associated with emotional and social stress in children as well as their parents [3, 5]. The concern is likely reflected in the common occurrence of referrals due to short stature as families wish for an accurate diagnosis and potential treatment [6]. Psychosocial assessment has been recommended as an integral component of evaluating patients with short stature [6, 7].

In a multinational consensus statement from 2008, short stature was seen as a possible drawback, and attaining an average height during childhood and in adult life was considered a meaningful medical achievement [2]. GH has received regulatory approval for ISS in multiple countries (United States 2003 and South Korea 2009); however, access to treatment remains uneven globally [8, 9]. The latest clinical treatment guidelines on ISS from the United States and Canada acknowledge that evidence remains insufficient to determine which surrogate markers beyond height should guide GH treatment decisions [10].

A systematic literature review, mainly reporting on children with GH deficiency (GHD), ISS, or short stature after being born small for gestational age, reported poorer quality of life (QoL) in short children compared to those with normal stature [5]. Children with comorbidities such as chronic kidney disease, achondroplasia, and transfusion-dependent b-thalassamia were naturally found to have the most impacted QoL; however, these patients substitute the minority of children treated with GH. Most children treated with GH have isolated GHD that is often idiopathic [11]. Psychological distress and lower QoL have been reported in children with idiopathic GHD and in a cohort of nonsyndromic children with various underlying causes to their short stature, including ISS [6, 12]. Research on the psychological consequences of ISS in children remains limited. Visser-van Balen et al [13] conducted a systematic review on psychosocial functioning of children with ISS, in which parents reported their child with short stature to have more psychosocial problems than their average-height peers. In another study, height-related psychosocial stressors were assessed in a Dutch adolescent population with ISS through parental surveys [14]. The sample size (n = 38), however, was insufficient to draw definitive conclusions on whether psychosocial concerns differed between adolescents with ISS and controls. The concern remains that short stature can contribute to various psychosocial issues, including social immaturity, feelings of being treated as a younger child, low self-esteem, and experiences of bullying [2].

The aim of this study was to assess the psychological burden of ISS by examining well-being as reported in mandatory questionnaires in school-aged children with ISS compared to a matched control group with normal height across the nation.

## Methodology

### Setting/Data sources

The Danish National Patient Registry (DNPR) maintains comprehensive records of all interactions that citizens have with hospitals and healthcare services that are publicly funded and free of charge. The records are maintained by automated surveillance. This includes in- and outpatient visits, scheduled and emergency admissions, and medical and surgical treatments. In- and outpatient contacts have been recorded since 1977 and 1995, respectively. Since 1968, every Danish citizen has been assigned by the Central Office of Civil Registration a unique 10-digit civil registration number by which all records can be linked across registries [15].

The Danish National Child Health Register was established in 2009, and in 2011 it became mandatory to record data [16]. Among many variables, the register contains heights for all Danish children. Midwives perform examinations of neonates and infants at home, and health nurses assess preschoolers, while general medical practitioners examine older children. During primary and lower secondary school, health nurses measure and record a child's height at least 3 times: twice during primary school and once during lower secondary school.

The National Well-being Survey, designed to obtain information on school well-being and subsequently to inspire improvements in the quality of the Danish public schools, was implemented in 2015. The questionnaire is based on work by an expert group and a pilot study [17]. It includes closed-ended questions that use a Likert scale to effectively measure students’ well-being and satisfaction and covers social well-being and academic performance. The survey is mandatory for all children in Danish public schools (grades 0 to 9) and is conducted annually. Two distinct questionnaires are applied: 1 for children in grades 0 to 3 (ages 6-9) and another for children in grades 4 to 9 (ages 10-15). For each question, children in grades 0 to 3 can respond with 1 of 4 possible answers: “yes,” “sometimes,” “no,” or “don't want to answer.” In contrast, children in grades 4 to 9 have a more granular set of options, allowing them to answer with “very often,” “often,” “sometimes,” “rarely,” “never,” or “don't want to answer.”

### Study design

#### Identification of children with ISS

This registry cohort study utilized individual data recorded in the National Child Health Register from 2012 to 2020 and evaluated heights against contemporary national growth charts while accounting for known causes of short stature. Included children had at least 1 height measurement below −2.0 SDS.

National growth charts developed by Tinggaard et al [18] were used to identify children with short stature. These charts describe the distribution of heights for boys and girls from 0 to 20 years within a reference population considered to be normal. The charts are based on quarterly references for children under 2 years of age and biannual references for children aged 2 to 20 years.

Two exclusion criteria were applied. The first criteria ensured the validity of the height measurements and was applied to the entire cohort. The second excluded children with ISS with any hospital contact potentially associated with an underlying cause of short stature. In the cohort of children with ISS, the following number of children were excluded:

1. Outliers. Height measurements identified as outliers were excluded from the analyses based on specific criteria indicating potential inaccuracies or atypical growth patterns. Measurements were considered outliers if a reduction of 10 cm or more compared to the previous measurement was observed (n children = 859, n measurements = 7346), if an increment of more than 40 cm in height within a period of less than 2 years (730 days) was observed (n children = 108, n measurements = 291), or if the height of an individual older than 5 years was less than 70 cm (n children = 8, n measurements = 21).
2. Comorbidities associated with short stature. This exclusion was based on International Classification of Diseases, Tenth Revision (ICD-10) codes recorded in the DNPR from 1995 to 2022. Children were excluded from the study if they had at least 1 hospital contact with a primary or secondary diagnosis code related to any of the conditions listed in Table 1 during this period. This exclusion criterion applied regardless of whether the diagnosis occurred before or after the child's inclusion in the study and thus reflects current practices in the public healthcare setting across the country that is free of charge for all citizens.

**Table 1.** ICD-10 codes potentially associated with short stature leading to exclusion of children

Reason to be excluded from the case population	ICD-10 code	Number of individuals excluded
Spinal abnormalities prohibiting accurate measure of stature	M41, M40	610
Unspecified behavioral syndromes associated with physiological disturbances and physical factors, eating disorders, other specified systemic involvement of connective tissue, chronic renal disease	F59, F50, M358, N18	399
Hormonal deficiencies	E23	572
Small for gestational age (birth length < −2.0 SD or birth weight < −2.0 SD or both)	P05 and P07	16 631
Cystic fibrosis	E86	837
Late puberty	E300	578
Diseases within the digestive system	K50, K51, and K52	425
Heart failure	“Heart failure” in register of chronic diseases (RUKS)	25
Congenital malformations, deformations, and chromosomal abnormalities	Q chapter	3376

Abbreviations: ICD-10, International Classification of Diseases, Tenth Revision; RUKS, Danish Register of Selected Chronic Diseases.

#### Study population

Children with ISS younger than 18 years were identified using age- and sex-specific height measurements below −2.0 and −2.5 SDS and were allocated into the primary and secondary case population, respectively. Children with a height measurement within −2.0 to +2.0 SDS constituted the control population. Each case was matched to 5 controls based on potential height-related confounders, including birth date, sex, residence region, parental education and income levels, and country of origin.

Well-being was assessed based on responses from the National Well-being Survey in the period from 2015 to 2022. The impact of ISS on well-being was estimated by comparing well-being in children with ISS to that of their matched control group.

#### Statistical analysis

The impact of ISS on well-being was assessed through a combination of linear regression and comparative analysis of survey responses. Linear regression was used to compare well-being between case individuals and their matched controls by estimating mean differences in the Likert score for each question included in the survey.

To assess responses made to questions related to central elements of well-being, such as whether respondents had felt bullied, lonely, or insecure, odds ratios (ORs) and their 95% confidence intervals (indicated in brackets) were calculated. The ORs compared the proportion of children with ISS answering negatively to the proportion of controls answering negatively. Analyses were conducted separately for grades 0 to 3, 4 to 6, and 7 to 9, reflecting the variations in the survey questionnaire across educational stages. Wald tests were conducted on the log-odds scale to compare the ORs from the primary and secondary case populations. A *P*-value below.05 was considered statistically significant.

## Results

**Figure 1.**
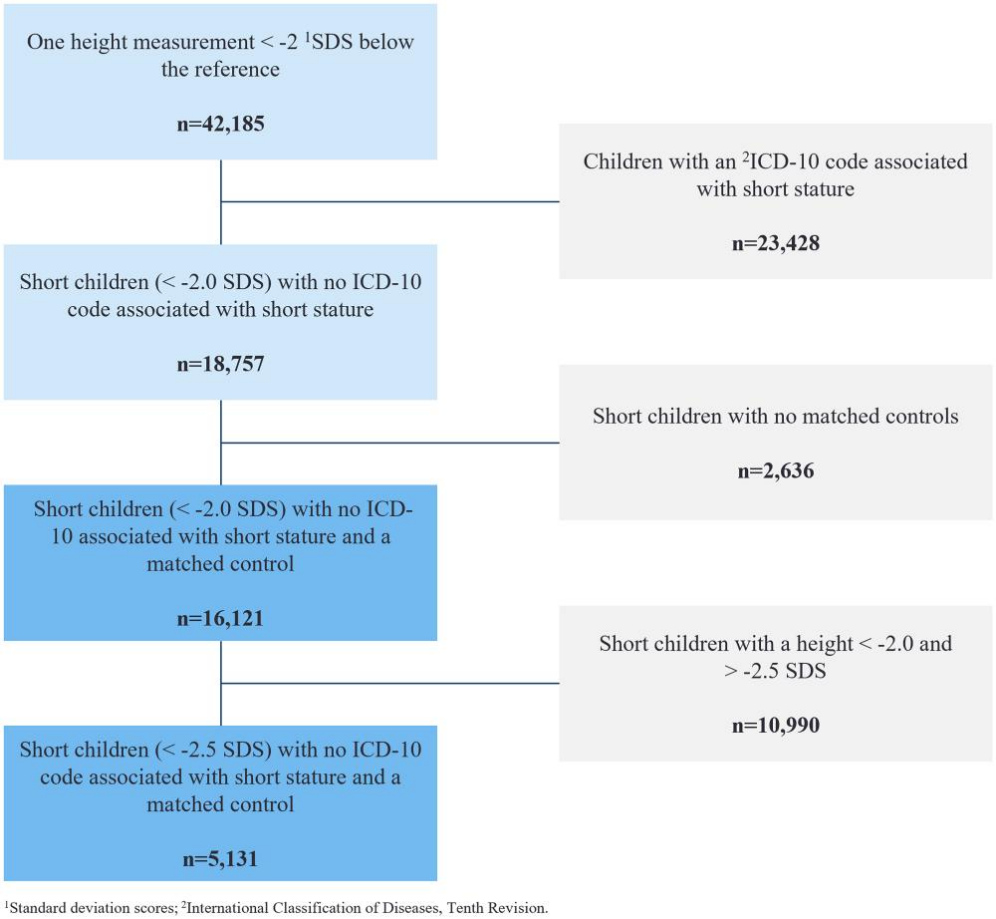
Flowchart showing the criteria applied to identify children with idiopathic short stature.

From the Danish National Child Health Register, 507 754 children with at least 1 height registration between 2012 and 2020 were identified. The number of children with at least 1 height measurements below −2.0 SDS and no outlier observations was 42 185. Among these children, a diagnosis code in the DNPR potentially associated with short stature was identified in 23 428, who were excluded from the analysis (Table 1). A matching control could not be identified for 2636 children, and they were also excluded. The resulting cohort, referred to as the primary case population, consisted of 16 121 children with an accompanying control population of 35 238 children (see Fig. 1). The prevalence of short stature with a height below −2.0 SDS was 3.2% (16 121/507 754) in the study period. Among the primary case population, 54% were boys (Table 2).

**Table 2.** Demographics

	Primary study population (height < −2.0 SDS)		Secondary study population (height < −2.5 SDS)	
	Case population: Children with ISS (%)	Control population: Children without ISS (%)	Case population: Children with ISS (%)	Control population: Children without ISS (%)
n	16 121	35 238	5131	11 654
Sex				
Male	8743 (54)	19 198 (54)	2780 (54)	6293 (54)
Female	7378 (46)	16 040 (46)	2351 (46)	5361 (46)

Abbreviations: ISS, idiopathic short stature; SDS, SD score.

In the primary case population (n = 16 121), 14 802 (92%) had more than 1 height measurement registered. An ICD-10 code describing short stature (E34.3) was recorded for 1991 children (12%) in the primary case population.

The subset of children in the primary case population with at least 1 height measurement below −2.5 SDS constituted the secondary case population (n = 5131). The prevalence of short stature below −2.5 SDS was 1.0% (5131/507 754) in the study period. In the secondary case population, 4773 (93%) had more than 1 height measurement registered. An ICD-10 code describing short stature (E34.3) was recorded for 1074 children (21%) in the secondary case population.

### Well-being

**Figure 2.**
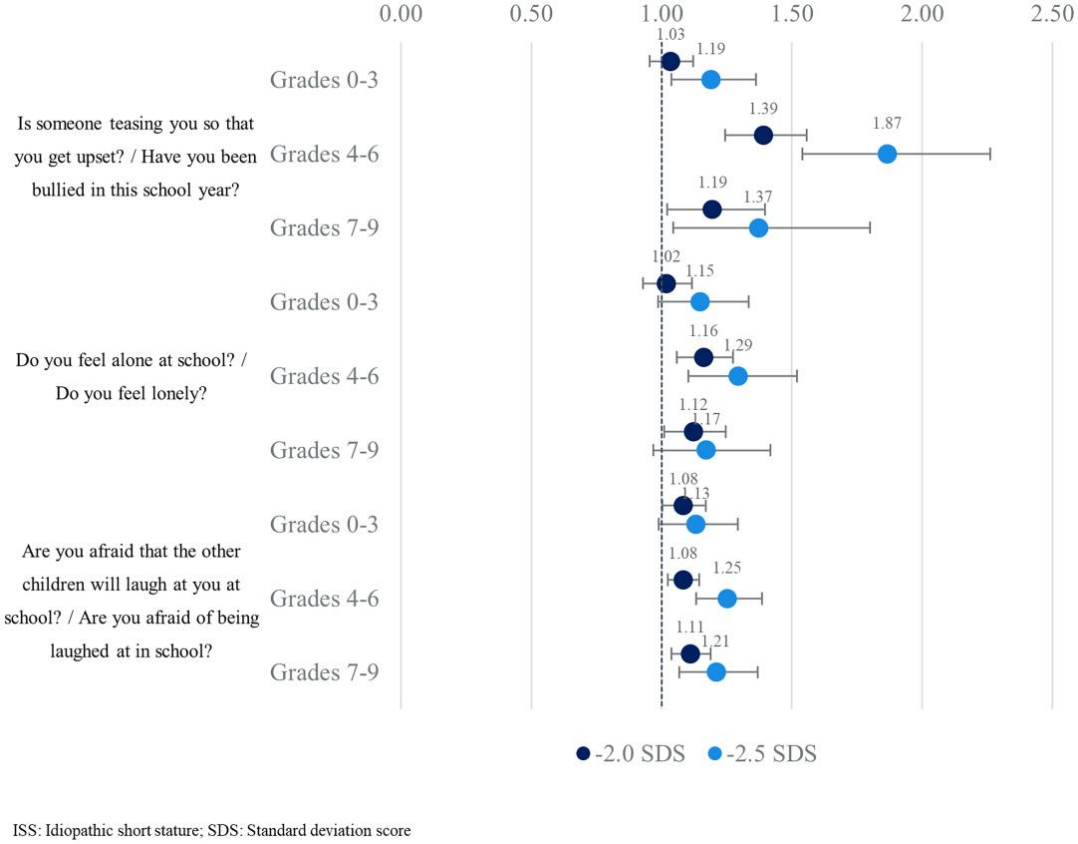
Forest plot showing odds ratios of responses to questions in the National Well-being Survey for children with idiopathic short stature (height < −2.0 SDS and < −2.5 SDS) with 95% confidence intervals stratified by grade. Abbreviation: SDS, SD score.

ORs comparing children from the primary (height <2.0 SDS) and the secondary (height < −2.5 SDS) case populations with their matched controls for selected questions from the National Well-being Survey are presented in Fig. 2. The numbers used for computation are presented in Table 3.

**Table 3.** Number of responders and ORs of responses to questions in the National Well-being Survey for children with idiopathic short stature (height < −2.0 SDS and < −2.5 SDS) with 95% CIs stratified by grade

Population	Outcome	Grade	No. answering the question		Percentage (%) answering “yes, often” or “yes, very often”		OR	95% CI
			Cases	Controls	Cases	Controls		
−2.0 SDS	Is someone teasing you so that you get upset?/Have you felt bullied during this school year?	0-3	12 623	19 821	8.5	8.3	1.03	[0.95-1.12]
		4-6	12 179	20 875	4.7	3.4	1.37	[1.24-1.56]
		7-9	9921	17 745	2.7	2.3	1.19	[1.02-1.40]
	Do you feel lonely at school?/Do you feel lonely?	0-3	12 768	20 240	6.2	6.1	1.02	[0.93-1.12]
		4-6	12 386	21 276	6.4	5.5	1.15	[1.06-1.27]
		7-9	10 000	17 875	6.1	5.4	1.11	[1.01-1.25]
	Are you afraid that the other children will laugh at you at school?/Are you afraid of being laughed at in school?	0-3	12 614	19 920	9.4	8.8	1.08	[1.00-1.17]
		4-6	12 349	21 218	19.6	18.4	1.07	[1.02-1.15]
		7-9	10 014	17 921	15.6	14.3	1.09	[1.04-1.19]
−2.5 SDS	Is someone teasing you so that you get upset?/Have you felt bullied during this school year?	0-3	4063	6673	9.7	8.3	1.17	[1.04-1.36]
		4-6	3669	6446	6.1	3.4	1.81	[1.54-2.26]
		7-9	3066	5606	3.0	2.2	1.36	[1.04-1.80]
	Do you feel lonely at school?/Do you feel lonely?	0-3	4115	6793	7.5	6.6	1.14	[0.99-1.33]
		4-6	3741	6548	7.4	5.8	1.27	[1.10-1.52]
		7-9	3068	5640	5.9	5.1	1.16	[0.97-1.42]
	Are you afraid that the other children will laugh at you at school?/Are you afraid of being laughed at in school?	0-3	4072	6684	9.9	8.8	1.19	[0.99-1.29]
		4-6	3729	6543	21.3	17.8	1.20	[1.13-1.39]
		7-9	3079	5652	15.5	13.2	1.18	[1.07-1.37]

Abbreviations: CI, confidence interval; OR, odds ratio; SDS, SD score.

Within the primary case population, children in grades 0 to 3 with ISS had higher odds of feeling bullied (OR 1.03 [0.95-1.12]), feeling lonely (OR 1.02 [0.93-1.12]), and feeling insecure (OR 1.08 [1.00-1.17]).

Children in grades 4 to 6 with ISS had significantly higher odds of feeling bullied (OR 1.37 [1.24-1.56]), feeling lonely (OR 1.15 [1.06-1.27]), and feeling insecure (OR 1.07 [1.02-1.15]).

Children in grades 7 to 9 with ISS had significantly higher odds of feeling bullied (OR 1.19 [1.02-1.40]), feeling lonely (OR 1.11 [1.01-1.25]), and feeling insecure (OR 1.09 [1.04-1.19]).

In the secondary case population (height below −2.5 SDS), the estimated ORs were amplified (Table 3). This was most pronounced for children in grades 4 to 6 where children with ISS had significantly higher odds of feeling bullied (OR 1.81 [1.54-2.26]), feeling lonely (OR 1.27 [1.10-1.52]), and feeling insecure (OR 1.20 [1.13-1.39]). Children in grades 4 to 6 in the secondary case population had a significantly increased risk of feeling bullied (*P* = 0.009) and feeling insecure (*P* = 0.013) compared to children in the primary case population.

The differences between children with ISS and their matched controls extended beyond the selected questions. Notably, children with ISS did not consistently provide lower scores than their matched controls, as evidenced by their higher satisfaction regarding school facilities (Table S1 [19] and Table S2 [20]).

The well-being of children with ISS was analyzed separately for boys and girls. Overall, boys and girls reported lower well-being compared to their matched controls. The odds varied slightly between sexes; girls reported a stronger feeling of having felt bullied while boys reported a stronger feeling of loneliness (Table S3 [21]).

## Discussion

In this nationwide register-based study, we identified that 3.2% of all children in Denmark fulfilled the criteria for ISS, corresponding to a height below −2.0 SDS, and that a smaller subgroup of 1.0% had a height below −2.5 SDS. Children with ISS experienced reduced well-being compared to children with normal height as measured by specific questions in the National Well-being Survey. Children in grades 4 to 6 more often felt bullied when compared to controls, and older children in grades 7 to 9 reported a stronger feeling of loneliness and insecurity compared to controls. Only in children with a height below −2.5 SDS were the findings statistically different from those in the control population. The OR of feeling bullied and insecure in children with a height below −2.5 SDS was statistically more pronounced than in children with a height < −2.0 SDS.

This study adds to the existing literature on the psychosocial burden of ISS by using a novel methodology that integrates various Danish register data sources. Although data were originally collected for another purpose, the data were systematically acquired, were of high completeness, and were relevant for the question at hand, supporting the reliability of the present findings. Our findings across the entire nation indicated that children with ISS have reduced psychosocial well-being compared to their peers of average height. González Briceño et al found that French children with nonsyndromic but other various underlying causes of their short stature including ISS had significantly lower QoL scores than the general pediatric population [6]. In that study, children with severe short stature (below −3.0 SDS) reported scores like or even less than those of chronically ill children [6]. Similarly, we identified, in a sensitivity analysis, that shorter stature (below −2.5 SDS) was strongly associated with reduced mental well-being in Danish schoolchildren. We did not examine if mental well-being differed between short children with an ICD-10 code referring to short stature (12% of the case cohort) and short children with an ICD-10 code with a relevant comorbidity compared to short children without these ICD-10 codes. A higher proportion of children with height below −2.5 SDS had a diagnosis code for short stature (21%), indicating hospital referral and assessment, which may reflect genuine clinical concern but could also primarily represent parental anxiety driving the request for assessment.

Children with ISS in our study were more likely to report experiences of bullying compared to the control group, and significance was reached in the shortest group of children with a height < −2.5 SDS. These findings are similar to those of Voss et al, who compared answers in a questionnaire on bullying between adolescent students who had been below the third height centile at school entry and a matched control group [22]. They found that shorter children were more susceptible to bullying than their taller peers and that many short students also reported feelings of social isolation [22]. Long-term consequences of childhood bullying are well documented, and children who experience bullying are at an increased risk for various mental, emotional, and behavioral issues later in life [23-25]. Given the seriousness of these implications, a thorough examination to identify potential underlying etiologies as well as a psychosocial assessment are essential to facilitate timely and relevant interventions. The question of which surrogate markers, beyond height, should inform the decision of relevant interventions in children with ISS in an era that emphasizes personalized medicine remains to be decided, particularly given that access to pharmacological treatment is not uniform across the globe [8, 9].

The literature on children with short stature is, in general, scarce and has varying results; many studies cannot confirm significant associations between short stature and QoL [26-30]. A Dutch study by Theunissen et al found that children with ISS scored lower in the social functioning domain compared to a control group but that children with ISS did not otherwise have reduced QoL [26]. A systematic review from 2021 concluded that children and adults with short stature of any cause may experience poorer QoL compared to those with normal stature but that further research is warranted [5]. The existing literature should be interpreted with caution, as many studies date back to the 1990s and early 2000s [27, 30-34]. This temporal gap raises concerns about the relevance of these studies, particularly considering the significant advancements in the diagnosis and treatment of ISS and short stature, as well as the increased societal acceptance of personal variance and expression over the past decades [4]. Measuring well-being and QoL in children presents various challenges, and many existing studies are hindered by the absence of control groups or by small sample sizes [35, 36]. Therefore, there is a critical need for more research to validate our findings to gain a deeper understanding of the experiences of these children and to examine the psychosocial impact of ISS.

Data on the impact of height on health-related QoL (HRQoL) in adulthood is scarce. A study conducted in the United Kingdom found that short stature in adulthood may be linked to a significant reduction in HRQoL [37]. In contrast, a French study suggested that height in adults was not associated with HRQoL; however, the study was conducted in the general population and not in a specific population of adults with short stature [38]. Future research should explore HRQoL within populations with short stature, preferably stratified by previous treatments, comorbidities, and gender.

### Strengths and limitations

A key strength of this study is the use of Danish national growth charts for identifying children with ISS as the charts provide a precise and standardized reference height tailored to specific ages. We identified short children using compulsory recordings of measurements registered within the Danish National Child Health Register across the country, minimizing the risk of selection bias. Robustness of the study was increased by the fact that 14 802 (92%) children had more than 1 height measurement recorded in the registry.

The absence of a specific ICD-10 code for ISS did not allow us to identify children via the DNRP; however, this approach would have overlooked children with short stature who had not presented for examination, as children would have had no ICD-10 codes registered. In our cohort, only 12% of children with a height below −2.0 SDS and 21% of children with a height below −2.5 SDS had an ICD-10 code for short stature. We attempted to omit children with a potential underlying diagnosis by excluding children with comorbidities according to ICD-10 codes in the DNPR associated with short stature regardless of the timing of any height recording (before or after). Yet we cannot exclude that some children were overlooked due to presentation with subtle clinical findings or did not present at all, resulting in the lack of an ICD-10 code (misclassification), which is an inherent risk when using epidemiologic data. We, however, feel this risk is minor as the cohort of ISS identified constituted 3.2% and thus approached the expected number of children with a height below −2.0 SDS at 2.1%.

Another strength of this study is the use of the National Well-being Survey, which is a validated tool for assessing well-being in Danish children. While it is a generic instrument, its widespread acceptance lends credibility to the findings. Nonetheless, it would be ideal to utilize a specific patient-reported outcome measure tailored to capture the unique aspects of HRQoL in children with ISS. Incorporating such specific measures in future research could enhance the sensitivity and relevance of the findings, providing a more nuanced understanding of the impact of ISS on well-being.

Children with ISS expressed a higher satisfaction regarding questions about school facilities, which serve as an important indicator of honesty in their survey responses and suggest that children are capable of providing genuine feedback, enhancing the credibility of the results. The ability to respond positively to questions unrelated to well-being alleviates concerns that children with ISS may have answered negatively across the board, thereby strengthening the validity of the reported well-being.

Participation in the National Well-being Survey is mandatory; however, children unable to participate on the survey day are not required to complete it later. If systemic differences in survey participation existed between children with ISS and those with average height, it could introduce bias, particularly if more children with ISS and reduced well-being were absent on the survey day. However, it is not anticipated that this factor substantially affected the results of the analysis, since children with ISS constituted 3.2%, which is just above the expected number of children with a height below −2.0 SDS (2.1%).

A final limitation of this study is that, due to its observational nature, we were unable to confirm a causal relationship between survey responses of well-being and height. We did not have direct contact with the children, which restricts our ability to assess the underlying factors influencing these data points. Nevertheless, the statistical significance of the observed correlation suggests a systematic trend that warrants further investigation.

## Conclusion

This nationwide cohort study identified that children with ISS have higher odds of experiencing bullying, loneliness, and insecurity compared to their peers of average height and that the tendency was more pronounced in the shortest children. The shortest children were also more likely to be registered with an ICD-10 code for short stature, indicating they had been examined in the hospital. The results emphasize the importance of identifying potential underlying causes of short stature and the need for psychosocial assessment to enable timely interventions. Further research is needed to identify which interventions may reduce the psychosocial burden in children with short stature and help identify which surrogate markers beyond height should guide clinical decision-making.

## Data Availability

Restrictions apply to the availability of some or all data generated or analyzed during this study to preserve patient confidentiality or because they were used under license. The corresponding author will on request detail the restrictions and any conditions under which access to some data may be provided.
